# Oxidative Stress in Neurodegenerative Diseases: From a Mitochondrial Point of View

**DOI:** 10.1155/2019/2105607

**Published:** 2019-05-09

**Authors:** Giovanna Cenini, Ana Lloret, Roberta Cascella

**Affiliations:** ^1^Institut für Biochemie und Molekularbiologie, University of Bonn, 53115 Bonn, Germany; ^2^Department of Physiology, Faculty of Medicine, University of Valencia, Avda, Blasco Ibañez, 17, 46010 Valencia, Spain; ^3^Department of Experimental and Clinical Biomedical Sciences, Section of Biochemistry, University of Florence, 50134 Florence, Italy

## Abstract

Age is the main risk factor for a number of human diseases, including neurodegenerative disorders such as Alzheimer's disease, Parkinson's disease, and amyotrophic lateral sclerosis, which increasing numbers of elderly individuals suffer. These pathological conditions are characterized by progressive loss of neuron cells, compromised motor or cognitive functions, and accumulation of abnormally aggregated proteins. Mitochondrial dysfunction is one of the main features of the aging process, particularly in organs requiring a high-energy source such as the heart, muscles, brain, or liver. Neurons rely almost exclusively on the mitochondria, which produce the energy required for most of the cellular processes, including synaptic plasticity and neurotransmitter synthesis. The brain is particularly vulnerable to oxidative stress and damage, because of its high oxygen consumption, low antioxidant defenses, and high content of polyunsaturated fats very prone to be oxidized. Thus, it is not surprising the importance of protecting systems, including antioxidant defenses, to maintain neuronal integrity and survival. Here, we review the role of mitochondrial oxidative stress in the aging process, with a specific focus on neurodegenerative diseases. Understanding the molecular mechanisms involving mitochondria and oxidative stress in the aging and neurodegeneration may help to identify new strategies for improving the health and extending lifespan.

## 1. Introduction

Aging is the primary risk factor for a number of human diseases, as well as neurodegenerative disorders [1], which increasing numbers of elderly individuals suffer. These pathological conditions, including Alzheimer's disease (AD), Parkinson's disease (PD), Huntington's disease (HD), amyotrophic lateral sclerosis (ALS), and spinocerebellar ataxia (SCA), are characterized by progressive loss of neuron cells, compromised motor or cognitive functions, and accumulation of abnormally aggregated proteins [2, 3]. A growing body of evidence highlights bioenergetic impairments as well as alterations in the reduction-oxidation (redox) homeostasis in the brain with the increasing of the age. The brain is composed by highly differentiated cells that populate different anatomical regions and requires about 20% of body basal oxygen for its functions [4]. Thus, it is not surprising that alterations in brain energy metabolisms lead to neurodegeneration.

Cellular energy is mainly produced via oxidative phosphorylation taking place within mitochondria, which are crucial organelles for numerous cellular processes, such as energy metabolism, calcium homeostasis, lipid biosynthesis, and apoptosis [5, 6]. Glucose oxidation is the most relevant source of energy in the brain, because of its high rate of ATP generation needed to maintain neuronal energy demands [4]. Thus, neurons rely almost exclusively on the mitochondria, which produce the energy required for most of the cellular processes, including synaptic plasticity and neurotransmitter synthesis [7]. Furthermore, given the central role of mitochondria in energy metabolism and in the regulation of the redox homeostasis, the study of age-related mitochondrial disorders is becoming nowadays of growing interest. Here, we review the role of mitochondria in the aging process, with a specific focus on mitochondrial oxidative stress in neurodegenerative diseases.

## 2. What Is the Oxidative Stress?

**Figure 1:**
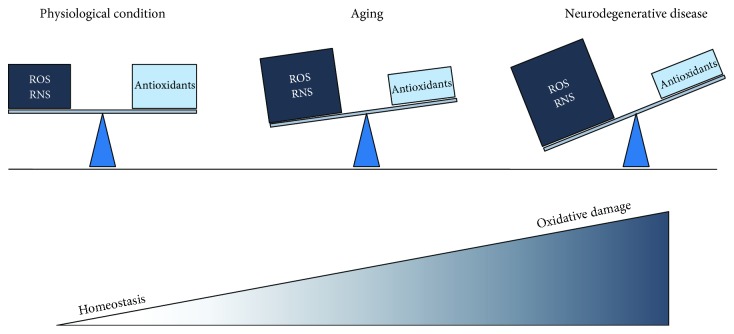
Schematic representation of oxidative stress in health, aging, and neurodegenerative diseases. In healthy conditions, the oxidant levels mainly produced in mitochondria are kept under control due to efficient mechanisms of defense that counterbalance the excessive production of oxidants and keep the homeostasis. However during the aging, the oxidant levels increase, while the antioxidant efficiency decreases generating an imbalance that leads to a noxious condition called oxidative stress and consequently to an oxidative damage of the main biomolecules such as proteins, lipids, nucleic acids, and carbohydrates. The overall picture intensifies in neurodegenerative conditions such as Alzheimer's disease, Parkinson's disease, and amyotrophic lateral sclerosis.

Reactive oxygen species (ROS) are normally produced in the cell of living organisms as a result of normal cellular metabolism and are fundamental in the maintenance of cellular homeostasis. In physiological conditions, low to moderate concentrations of ROS are involved in processes such as immune response, inflammation, synaptic plasticity, learning, and memory [8]. However, the excess of ROS production can be harmful, producing adverse oxidative modifications to cell components including mitochondrial structures as the first targets of ROS-induced damage [9]. Nevertheless, the human body is equipped with a variety of antioxidants that serve to counterbalance the effect of oxidants, including superoxide dismutase (SOD) and the glutathione (GSH) system [10]. When an imbalance between free radical production and detoxification occurs, ROS production may overwhelm antioxidant defenses, leading to the generation of a noxious condition called oxidative stress and overall to the impairment of the cellular functions. This phenomenon is observed in many pathological cases involving mitochondrial dysfunction, as well as in aging [11] (Figure 1). The brain is particularly vulnerable to oxidative stress and damage, because of its high oxygen consumption, low antioxidants defenses, and high content of polyunsaturated fats very prone to be oxidized [12].

Biological molecules such as proteins, lipids, nucleic acids, and carbohydrates are generally prone to oxidation, leading to a consistent oxidative damage of the biomolecules like change of their structures and consequently to their functions. The resulting oxidative modifications of the biomolecules are quite stable and they could be used as markers of oxidative and nitrosative stress. For example, the main products of protein oxidation are protein carbonyls and nitrated proteins [13]. Protein carbonyls derive by the direct oxidation of certain amino acids by peptide backbone scission or by Michael addition reaction with products of lipid peroxidation (e.g., HNE) or by glycoxidation reactions [14]. Detoxification of protein carbonyls happens through enzyme such as aldehyde dehydrogenase (ALDH) or by reduction of the carbonyl group to the corresponding alcohol group by carbonyl reductase (CR) [15]. Protein nitration happens in particular at tyrosine level (3-nitrotyrosine: 3-NT) through the action of reactive nitrogen species (RNS) such as peroxynitrite and nitro dioxide [16].

Another characteristic process of oxidative stress that affects lipids and leads to the formation of the relative markers is the lipid peroxidation. More in specific, lipid peroxidation derives from the damage of cellular membranes by ROS that generates a heterogeneous group of relatively stable end-products such as malondialdehyde (MDA), 4-hydroxy-2-nonenal (HNE), acrolein, and isoprostanes [17]. MDA, HNE, and acrolein are able to bind proteins and DNA leading to the alteration of conformation and function [18].

Carbohydrates are also affected by ROS. Indeed, reducing sugars plays a pivotal role in modifying proteins through the formation of advance glycation end-products (AGEs) in a nonenzymatic reaction called glycation [19]. AGEs are involved in the progress of some diseases such as diabetes mellitus, cardiac dysfunction, and neurodegenerative diseases [20].

Between all the free radicals, the hydroxy radical (OH^·^) is the most toxic because of its high reactivity and limitation on its diffusion from their site of the formation. OH^·^ has been found to damage biological molecules including nucleic acid [21]. 8-Hydroxyguanosine (8-OHG) and 8-hydroxy-2′-deoxyguanosine (8-OHdG) are the most abundant among the oxidized bases, and they can be used as markers of RNA and DNA oxidation [22]. The involvement of nucleic acid oxidation in neurodegenerative diseases might cause not only the reduction of protein level but also translation errors *in vivo* with alteration of protein structure and function [23].

In the course of the evolution, the organisms have developed several mechanisms of protection against the noxious effects of ROS and RNS in such a way that the whole amount of prooxidants is under control, and the negative consequences are limited. The antioxidant molecules are divided into two groups: enzymatic and nonenzymatic compounds. The enzymatic group includes superoxide dismutase (SOD), catalase (CAT), glutathione peroxidase (GPx), and glutathione reductase (GR). SOD is one of the first protective mechanisms against ROS and catalyzes the conversion of O2^**-·**^ to H_2_O_2_ and O_2_ [24]. The generated H_2_O_2_ is converted to water and O_2_ by CAT. The nonenzymatic group involves glutathione (GSH), the most abundant antioxidant in most of the brain cells, thioredoxin (Trx), vitamins A, E, and C, and selenium. GSH reacts with ROS generating glutathione disulfide (GSSG) and enters a cycle together with GPx and GR. Vitamin E (also called *α*-tocopherol) is a lipophilic molecule acting against the lipid peroxidation [25]. Vitamin C (also called ascorbic acid) is one of the most important water-soluble antioxidants. Selenium is a crucial cofactor for the enzymes GPx and thioredoxin reductase (TrxR) and essential trace elements. All together they act and balance the levels of ROS and RNS to avoid the onset and the propagation of harmful effect in the nearby tissues.

## 3. Mitochondrial Damage in Aging

Aging is a degenerative physiological process induced by the accumulation of cellular lesions leading progressively to organ dysfunction and death. Although our knowledge of the aging process remains far from being complete, understanding the basis of human aging is one of the great biomedical goals. The best known and most long-standing hypothesis to explain aging is the “free radical theory of aging” proposed by Harman and coworkers [26], which postulates that aging and age-associated degenerative diseases are the result of free radical attacks on cells and tissues. This theory was later extended by Miquel and coworkers [27] who focused on mitochondria as the main source of ROS in aging cells. In this relevant work, the authors explained how mitochondrial disorganization might be an important aspect of the age-related changes of postmitotic cells such as neurons and muscle cells. This view was based on electron microscopic and biochemical studies on insects and mammals. Finally, they offered a hypothesis on intrinsic mitochondrial senescence and its possible relation to age-related changes in other cell organelles. The theory is known nowadays as “the mitochondrial theory of aging.” Since this early publication, experimental evidences of the implication of mitochondria in aging have increased.

**Figure 2:**
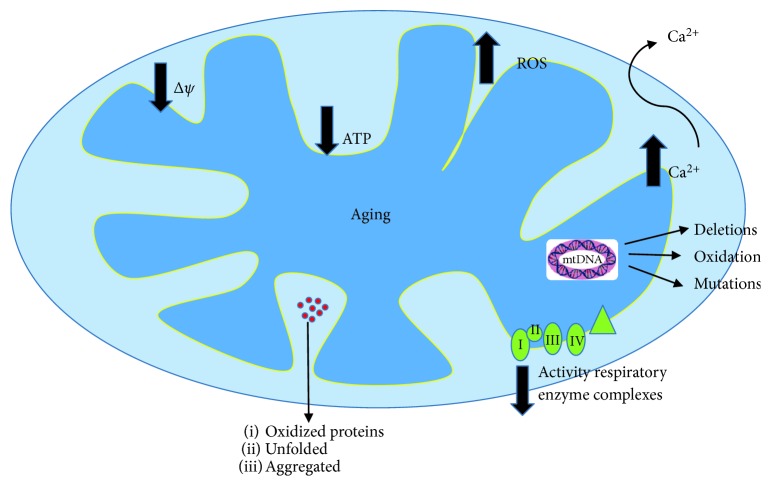
Rendition of the central role of mitochondrial deficiencies in aging. The ROS production in aged mitochondria is increased, the membrane potential appeared lower, ATP synthesis is reduced, the activity of respiratory enzyme complexes is declined, and oxidized proteins accumulate causing protein aggregation. Mitochondrial DNA (mtDNA) is also oxidized and deletions and mutations have found.

In addition to the energy generation through oxidative phosphorylation, mitochondria play an essential role in cell metabolic homeostasis, signaling, differentiation, and senescence [28]. Mitochondrial dysfunction is one of the main features of the aging process [29] (Figure 2), particularly in organs requiring a high-energy source such as the heart, muscles, brain, or liver. Although a large amount of data support the role of mitochondrial ROS production in aging, it has also recently been demonstrated the involvement of the mitochondrial permeability transition in the mechanisms of aging [30]. Indeed, the mitochondrial membrane potential appeared originally lower in old animals, and cellular peroxide levels were higher in cells from old animals with respect to the young ones [31]. The age-associated decrease in mitochondrial membrane potential correlated with reduced ATP synthesis in tissues of old animals [32] and also in human fibroblasts from elderly subjects [33]. The mitochondrial permeability transition is due to a nonspecific pore called the mitochondrial permeability transition pore (mPTP) occurring when mitochondria become overloaded with calcium. Indeed, it is well known that aging alters cytosolic calcium pick-up and the sensitivity of the mPTP to calcium enhanced under oxidative stress conditions [34].

Using isolated mitochondria, in the past few decades, many studies revealed that the activity of respiratory enzyme complexes in the electron chain transport gradually declines with age in the liver, skin fibroblasts, brain, and skeletal muscle of humans [32, 33, 35, 36] (Figure 2). Moreover, mitochondrial morphology changed with age. Electron microscopic studies showed that mitochondrial disorganization accumulates with age in a variety of cells and tissues [26]. Although mitochondria are very dynamic organelles and can remodel their structure through fusion and fission [37], abnormalities in the process have been related to senescence in mammalian cells [38].

Age-associated oxidative damage to mtDNA was shown to correlate with mitochondrial GSH oxidation in the liver, kidney, and brain of rats and mice [39]. mtDNA deletions were also found to correlate with the level of oxidized guanosines in mtDNA [40]. Furthermore, mtDNA increasingly accumulated mutations with age in a variety of human tissues, which include point mutations [41], large-scale deletions [42], and also tandem duplications [43] (Figure 2). On the other hand, mitochondrial rRNAs were oxidized and degraded under oxidative stress conditions [44]. Oxidized proteins also accumulate progressively during aging, and an important consequence is the unfolding phenomenon that causes protein aggregation [45] (Figure 2). Many respiratory enzymes are known to be the targets of oxidation, such as complex I and ATPase [46] (Figure 2). Finally, lipid peroxidation is particularly important in the inner mitochondrial membrane due to the high content of cardiolipin [47]. In fact, oxidative stress was found to decrease cardiolipin levels more than other lipids and this decline appeared directly related to the decrease of cytochrome oxidase activity [47].

Interestingly, a switch from glycolysis to respiratory metabolism in yeast has been found to increase ROS production, activate the antioxidant response, and increase NADPH production, causing lifespan extension and hormesis response [48].

Mitochondrial dysfunction has also been related to another aging-related process, the telomere shortening [49]. PGC-1a/b are the principal regulators of mitochondrial biogenesis and function and establish the connection between telomere shortening and mitochondria malfunction [50]. When DNA is damaged, p53 levels increase and PGC1a/b are inhibited consequently leading to mitochondrial dysfunction [49, 50]. PGC-1a was also found to decrease its activity inducing loss of SIRT1 activity and mitochondrial dysfunction, particularly in the muscle [51]. Interestingly, the overexpression of PGC-1a can improve aging muscle and plays a significant role in longevity [52]. On the other hand, DNA damage can also activate AKT and mTORC1, resulting in PGC-1b-dependent mitochondrial biogenesis and ROS generation [53].

## 4. Mitochondrial Oxidative Stress and Its Role in Neurodegenerative Diseases

As already described above, mitochondria are key multifunctional organelles that play multiple important functions in the cell. They are essential not only in energy production but also in thermogenesis, calcium homeostasis, generating and maintaining key cellular metabolites, and redox signaling [5]. Neurons are postmitotic highly differentiated cells with a lifespan similar to that of the whole organism [54]. These excitable cells are more sensitive to the accumulation of oxidative damages compared to dividing cells and are more prone to accumulating defective mitochondria during aging [54, 55]. Thus, it is not surprising the importance of protecting systems, including antioxidant defenses, to maintain neuronal integrity and survival.

All the neurodegenerative disorders share several common features, such as the accumulation of abnormally aggregated proteins and the involvement of oxidative damage and mitochondrial dysfunction. Many of the genes associated with PD or ALS are linked to mitochondria. In addition, all aggregated misfolded proteins (*β*-amyloid, tau, and *α*-synuclein) are known to inhibit mitochondrial function and induce oxidative stress [56]. Therefore, the identification of common mechanisms underlying neurodegenerative diseases, including mitochondrial dysfunction, will increase our understanding of the essential requirements for neuronal survival that can inform future neuroprotective therapies.

## 5. Alzheimer's Disease

Alzheimer's disease (AD) is the most prevalent neurodegenerative disorder affecting the aged population, which is characterized by progressive deterioration of behavior, cognition, and functionality [57]. Although the pathophysiology is extremely complex and heterogeneous, the main hallmarks of AD are the senile plaques composed by extracellular deposition of amyloid beta (A*β*) peptide and the presence of intracellular tau neurofibrillary tangles (NFT) [57]. The aberrant protein aggregation results in multifactorial neuronal dysfunction affecting synaptic signaling, mitochondrial function, neuroinflammation, and neuronal loss [58, 59]. In particular, A*β* plaques were found to deplete Ca^2+^ ions storage in the endoplasmic reticulum (ER), resulting in cytosolic Ca^2+^ overload, which causes a reduction in endogenous GSH levels and ROS accumulation [60]. ROS-induced oxidative stress is one of the main important factors in the pathogenesis of AD as ROS overproduction is thought to play a critical role in the accumulation and deposition of A*β* peptides in AD [61].

The relationship between mitochondria and AD pathology is not so direct compared to other neurodegenerative disorders, although the role of oxidative stress and mitochondrial dysfunction is shown in different AD models [62]. Thus, a reduction in complex IV activity has been demonstrated in mitochondria from the hippocampus and platelets of AD patients and in AD cybrid cells [63, 64]. Aggregation of A*β* peptides leads to oxidative stress, mitochondrial dysfunction, and energy failure prior to the development of plaque pathology [65] and can reduce mitochondrial respiration in neurons and astrocytes via the inhibition of complexes I and IV, thus causing ROS production [66]. The important role of mitochondrial ROS has been also confirmed by the results obtained with the antioxidant MitoQ, which can prevent cognitive decline, A*β* peptide accumulation, microglia inflammation, and synaptic loss in a transgenic mouse model of AD [67] and can extend lifespan and improve health in a transgenic *Caenorhabditis elegans* model of AD [68]. In addition, the inhibition of oxidative stress as a result of a polyunsaturated fatty acid diet improved cognition and memory in mice [69], rats [70], and protected worms from the paralysis by extending their lifespan [71].

It has been reported that the H_2_O_2_ production from synaptic mitochondria was more than fivefold higher than that from nonsynaptic mitochondria [72]. This fact indicates that neurons are more susceptible to oxidative damage than glial cells. Furthermore, isolated mitochondria from neurons incubated with A*β* peptides caused a fivefold increase in the rate of H_2_O_2_ production [73]. Moreover, A*β* peptides increased the aggregation of mitochondria isolated from neurons and caused cytochrome C release from mitochondria, both proapoptotic signals [73]. Studies performed in AD patients showed reduced cytochrome oxidase activity in platelets of AD subjects when compared to controls [74], and this mitochondrial defect was also demonstrated in the brain of AD patients [75]. Mitochondria from platelets of AD patients have been found depolarized, smaller on average, and less able to buffer calcium, showing lower ATP levels and an increase of oxidative stress, stress signaling, and apoptosis, with respect to controls [7]. Subjects with mild cognitive impairment also revealed mitochondrial deficiencies [76]. Fisar et al. have recently shown that both insufficiency in substrates entering into the oxidative phosphorylation system and functional disturbances in the electron transport system complex are responsible for the decrease in respiration observed in intact platelets of AD patients [77]. A very early decrease in mitochondrial complex activity has also been found in the entorhinal cortex of AD patients, but not in the frontal cortex [78].

Recently, it has been suggested that an imbalance in nuclear and mitochondrial genome-encoded oxidative phosphorylation transcripts may drive a negative feedback loop reducing mitochondrial translation and compromising oxidative phosphorylation efficiency, leading to ROS production [79]. Indeed, a deficiency of the mitochondrial oxidative phosphorylation system can impact directly on mitochondrial function and result in several disease phenotypes [80].

It has recently been shown that cells from late-onset AD patients exhibited an impaired mitochondrial metabolic potential and an abnormal redox potential, associated with reduced nicotinamide adenine dinucleotide metabolism and altered citric acid cycle activity [81]. Moreover, AD fibroblasts presented a significant reduction in mitochondrial length, changes in the expression of proteins that control mitochondrial fusion, and dysfunction of mitochondrial bioenergetics [82]. Martín-Maestro et al. also showed that multiple genes that control mitochondrial homeostasis, including fission and fusion, are downregulated in Alzheimer's patients. These defects lead to strong accumulation of aged mitochondria in AD fibroblasts [83]. Accordingly, the analysis from AD patients of genes involved in autophagy and mitophagy demonstrated a downregulation, indicating that the recycling mechanism of these aged mitochondria might be impaired [83].

AD pathogenesis has also been linked to voltage-dependent anion channel 1 (VDAC1) [84], which is expressed in the mitochondrial outer membrane and regulates the main metabolic and energetic functions of the cell, including Ca^2+^ homeostasis, oxidative stress, and mitochondrion-mediated apoptosis. Indeed, VDAC1 levels were found to increase in the AD brains and its inhibition has been proposed as the target of a novel strategy for diminishing cell death [84].

Mitochondria are highly abundant in synapsis cause of their on-site energy provision and calcium modulation [85]. Via TOM import machinery [86] and/or by local production by *γ*-secretase [87], A*β* peptides accumulate inside the synaptic mitochondria [88]. Then, it probably interacts with the mitochondrial heme group [73] and/or with mitochondrial matrix proteins such as amyloid-binding alcohol dehydrogenase (ABAD) [188] and blocks the electronic transport thereby compromising ATP production [89] and synaptic function [64]. A recent study performed in isolated mammalian mitochondria showed that A*β* peptides impaired mitochondrial import of nuclear-encoded precursor proteins due to a coaggregation process [90].

It has been showed from different laboratories that oxidative and nitrosative stress markers were substantially increased in AD, encouraging the idea about the crucial role of these two pathways in the progression of the disease. In several postmortem cortex areas not only from sporadic and familiar AD patients but also from patients affected by mild cognitive impairment (MCI), the levels of protein carbonyls were substantially increased compared to age-matched control subjects [91, 92]. In addition, the levels of carbonyl reductase (CR) were also found to increase in the AD brains, suggesting that the brain tries to counteract protein oxidation [15]. About markers of nitrosative stress in AD, proteomic approaches have identified a large number of proteins which are nitrated in the MCI and AD brains [93, 94]. These proteins are involved in several cellular functions such as energy metabolism, structural maintenance, pH regulation, and antioxidant.

Lipid peroxidation seems to play a particular role not only in aging but also in the pathogenesis of AD [95] and its products could be used as markers for AD identification since early stages (MCI). Indeed, in CSF and the brains from AD and MCI subjects, it has been found elevated levels of lipid peroxidation products such as HNE, malondialdehyde (MDA), acrolein, F_(2)_-isoprostane, F_(4)_-isoprostane, and neuroprostane [96, 97], have been found elevated, while MDA levels was also found high in plasma and serum from AD patients [98] and colocalized with neurofibrillary tangles and senile plaques [99]. The decrease of the detoxification system efficiency in MCI and AD caused the accumulation of HNE protein adducts in neuronal cells [100]. Also in the blood, a significant increase of HNE levels has been found in AD patients compared to healthy subjects [101]. Acrolein has been reported to react with DNA bases leading to the formation of acrolein-deoxyguanosine in the AD brain [102].

Several papers published in the early nineties suggested an important role of glycation in the formation of neurofibrillary tangles and senile plaques [103, 104]. Like many other markers of oxidative stress, AGEs were also found to increase in CSF of AD patients, as well as their receptor levels in microglia cells of the AD brains [104]. While the picture in the brain is clear, the results about AGEs and soluble RAGE levels obtained in the blood from AD patients are controversial [105].

A considerable amount of evidences supports the early involvement of nucleic acids and oxidation in the cascade of neurodegeneration. DNA and RNA damage is a feature of the AD brain as well as of peripheral tissue [106]. mtDNA of cortical neurons in AD patients was found deleted with respect to age-matched controls [107]. Later, sporadic mutations in the mtDNA control regions in AD patients were also identified [108]. Interestingly, 8-OHdG levels in the mtDNA of the cerebral cortex and cerebellum from AD patients were threefold higher than in age-matched controls [109]. RNA oxidation was observed in the postmortem brains of early and latest stages of AD [110], a presymptomatic case with familial AD mutation [111], and Down syndrome with AD pathology [112], suggesting that mRNA is highly sensitive to oxidative damage. Fivefold increase in oxidized RNA was also observed in CSF of AD cases [113]. All this data suggests that nucleic acid oxidation may be considered an early event in the progression of AD.

The antioxidant levels in AD were found to change not only in the brain but also in peripheral tissues. Most of the studies have found an overall decrease in antioxidant amount and activity in the blood of AD patients since the early phases, suggesting that the physiological equilibrium between ROS/RNS production and antioxidant is altered and consequently the amount of antioxidant available is strongly compromised. In particular, SOD levels, but not the activity [114], were found to be elevated in the hippocampus and amygdala of AD patients [115], while a decrease in SOD, GPx, and CAT levels was found in the frontal and temporal cortex [116]. Nevertheless, CAT activity was found to increase in AD erythrocytes [117], suggesting an independence of the redox status between the periphery and the brain.

GSH was also found to decrease in the MCI and AD brain and erythrocytes [114, 118]. Another recent study showed higher GSH levels in the anterior and posterior cingulated from MCI patients [119]. In addition to GSH, the enzymes involved in its metabolism were also analyzed. In particular, glutathione-S-transferase (GST), a sensitive target of oxidative and nitrosative stress, was found to be carbonylated in *C. elegans* expressing A*β*42 [120] and in canine model of aging [121]. GST was also found to be nitrated in inferior parietal lobe (IPL) from MCI patients [122] and significantly elevated in the AD hippocampus, causing a decrease of its activity [118]. Since GSTs catalyze the conjugation of HNE to glutathione (GSH), the decline of its activity consequently leads to the compromise of detoxification process of HNE [123] and an accumulation of HNE-modified proteins. All these studies suggest that an alteration of GSH metabolism at the early stages of the disease could be an early marker for the detection of AD.

Another family of antioxidants particularly affected by oxidative and nitrosative stress is peroxiredoxins (Prxs), which reduces H_2_O_2_ [124] and presents a redox-regulated chaperone activity [125]. Prx2 oxidation was found A*β*42 dependent in SAMP8 mice [126]. However, Prx2 expression was found to increase in the AD brains [127] and Prx6 is oxidatively modified in the MCI brains [122], suggesting the presence of a compensatory mechanism.

Vitamins E and C were also found to decrease in plasma from MCI and mild AD and in CSF from AD patients [128–130]. In line with these results, another study showed a positive correlation between plasma vitamin E levels and the risk to develop AD in an advanced age [131].

Selenium levels were also affected in AD plasma with an association to the cognitive decline [132]. Nevertheless, the plasma levels of selenium seem to be independent from those of the brain [133]. The levels of seleno-containing enzyme Trx1 were also found to increase in the AD brains [134], in particular in glial cells, but not in neurons [135]. On the contrary, the long cleavage product of Trx1, Trx80, was drastically reduced in the brains and CSF from AD and MCI patients and it could be used to distinguish the stable MCI from the MCI that evolve later to AD [136].

## 6. Parkinson's Disease

Parkinson's disease (PD) is the second most prevalent neurodegenerative disorder, after AD, which is characterized by the progressive degeneration of the dopaminergic neurons located in the substantia nigra (SN) pars compacta [137]. The main neuropathological hallmark of PD is the presence of intracellular inclusions known as Lewy bodies (LBs) and neurites (LNs) [138], predominantly composed by misfolded and aggregated forms of the presynaptic protein *α*-synuclein [139].

The implication of mitochondrial dysfunction in the pathology of PD has been shown for a long time [140, 141]. The aging-related mitochondrial decline and the increasing mtDNA damage/mutations are also been associated with the increased risk for PD [142]. Indeed, it has been reported that mtDNA can impair the capacity of the organelle quality control mechanisms and thereby amplify the initial insult through a progressively increase of metabolic dysfunction and oxidative stress [143]. In particular, in some models of PD, it has been shown that environmental xenobiotics were extremely toxic and caused mitochondrial dysfunction in dopaminergic neurons leading to parkinsonian phenotypes [144].

Furthermore, the protein *α*-synuclein associated with PD pathogenesis is known to target the mitochondria and to decrease their function [140–142, 145, 146]. Notably, a decline of complex I activity and elevated intracellular ROS have been reported in the SN of the postmortem brain of PD patients [140, 147]. The implication of the mitochondria in PD is also supported by the presence of PD-related genes such as PINK1, PARK2 (Parkin), DJ-1, and LRRK2 which regulate mitochondrial and ROS homeostasis [148–151]. PINK1 deficiency results in impaired respiration with inhibition of complex I, and mutations in PINK1 gene cause a recessive form of PD [152, 153]. Abnormal ROS production in the mitochondria of PINK1 knockout neurons has been found to inhibit the mitochondrial Na^2+^/Ca^2+^ exchanger or glucose transporter and to be prevented by antioxidants [152, 154]. Mitochondrial ROS play an important role not only in the pathology of PINK1 (mutation or deficiency) but also in the physiology of PINK1/Parkin-related mitophagy, by the induction of mitochondrial recruitment of Parkin [155]. In addition, it has recently been reported that mitochondrial ROS production in familial and sporadic forms of PD caused DNA damage and activated the PARP enzyme-associated DNA repair mechanism [156].

Although monomeric *α*-synuclein is a physiological regulator of synaptic transduction and mitochondrial bioenergetics [157], the oligomeric species appeared toxic for cells [62, 158], inhibited mitochondrial complex I [159], and induced mitochondrial depolarization [160]. In addition, oligomeric *α*-synuclein caused ROS production [158] independently of the known enzymatic pathways that affected mitochondrial function and induced lipid peroxidation [161]. The role of *α*-synuclein in mitophagy, mitochondrial fission/fusion, and protein trafficking to this organelle has also been shown [162]. Neurodegenerative impairments were also found in *α*-synuclein transgenic mice through the activation of mPTP [163]. The opening of mPTP appeared to be induced by oligomeric *α*-synuclein with respect to the monomeric protein, due to their ability to induce calcium signal in a structure-specific manner [164] and to produce ROS in the presence of free metal ions [165].

In addition to the mitochondrial dysfunction and ROS production, PD is characterized by an overall increase of end-product markers of oxidative and nitrosative stress that reflects an extensive damage on the biomolecules. This strongly suggests that these three processes are interconnected and create a cascade of events promoting a neurodegenerative condition like PD. The SN from the postmortem brains showed increased protein carbonyl levels at high molecular weight compared to the control brains or other brain regions [165]. The levels of protein nitration were also found to increase in the PD brains [166]. A recent study showed increased *α*-synuclein nitration levels in the brain from individuals with synucleinopathy, suggesting a direct link between nitrosative damage and the progression of neurodegenerative synucleinopathies [167]. Interestingly, an *in vitro* study showed the nitration of mitochondrial complex I that might trigger overtime a cascade of deleterious events enhancing the overall oxidative damage in PD [168]. The postmortem brains of PD patients showed increased levels of lipid peroxidation markers and oxidized proteins [169]. In particular, HNE adducts were identified in dopaminergic cells of SN, CSF, and plasma from PD patients [170, 171] and significant differences were found in plasma of PD subjects treated with L-dopa therapy [171]. Moreover, MDA levels were found to be high and attached to *α*-synuclein in the SN and frontal cortex of PD cases [172] supporting the idea that lipid peroxidation might precede and contribute to *α*-synuclein aggregation. In PD plasma, MDA levels were also found to be increased and inversely related to the age of patients [173]. Glycation also showed a strong immunoreactivity in the SN and locus coeruleus of PD patients [174], suggesting its involvement in the chemical cross-linking, proteolytic resistance, and aggregation process. Overall, the levels of glycated proteins were significantly higher in the cerebral cortex of PD patients compared to age-matched controls [175]. Since in PD the catabolic pathway activity of the most glycation agents is lower [176], the AGE concentration rises up leading to the death of dopaminergic neurons. About DNA and RNA oxidation, the levels of 8-OHdG and 8-OHG were also found to be increased in different brain regions, including SN, in serum and CFS [177, 178].

The antioxidant status of PD was found significantly modified compared to age-matched healthy subjects, as well as in AD. In particular, SOD levels, but not the activity, were found to increase in the SN and basal ganglia from PD patients [179]. In contrast, the levels of other antioxidant enzymes such as CAT, GPx, and GR did not change in PD compared to the age-matched healthy subject [179]. GSH levels were found to decrease only in the SN [180]. Interestingly, blood GSH/GSSG ratio was found to increase when the patients stopped to take PD medication such as dopamine receptor agonist suggesting how the medication could strongly influence the peripheral redox status [181].

Anyway, studies in the animal models of PD showed different outcomes compared to the human sample analysis with a degree of controversy [182, 183] probably due to the temporal length of the experimental observation time.

## 7. Amyotrophic Lateral Sclerosis

Amyotrophic lateral sclerosis (ALS) is a neurodegenerative disease characterized by progressive loss of motor neurons in the anterior horn of the spinal cord, leading to muscle weakness, wasting, and spasticity [184]. ALS is classified as either familial or sporadic depending on whether there is a clearly defined, inherited genetic element. The onset of sporadic (sALS) is unknown, and thus, the identification of causal genes and environmental factors remains elusive. Mutations in the first ALS gene superoxide dismutase 1 (SOD1) were found in 1993 to segregate in several fALS pedigrees [185] and subsequently in a small number of unrelated sALS cases [186]. Fifteen years later, TAR DNA binding protein 43 (TDP-43) is found to be an important constituent of protein aggregates frequently observed in postmortem material of ALS patients [187]. Although it is not yet clear how such aggregates trigger neurodegeneration in ALS, mutations in the TDP-43 gene were reported in 3% of fALS and 1-5% of patients with sALS, suggesting that TDP-43 aggregates have a central role in triggering ALS [188, 189]. The number of ALS genes increases and it appears that the mutant proteins encoded are involved in a variety of critical processes, including mitochondria function.

The link of mitochondria to ALS has been defined by the mutations in SOD1 gene since they were found in 20% of the fALS [190]. SOD1 is an ubiquitous enzyme with several functions, including scavenger of excessive superoxide radical (O_2_^–·^) into oxygen, modulation of cellular respiration, energy metabolism, and posttranslational modification [191]. Several SOD1 mutations were shown to affect the folding of the protein, and it is believed that the ensuing toxic gain of function might be caused by the accumulation of misfolded proteins inside the intermembrane space of mitochondria [192] with generation of free radicals that eventually lead to cell injury and death [193, 194]. Even if this may not be the main triggering mechanism, it is nonetheless recognized that mitochondrial dysfunction is central to SOD1 pathogenesis [195]. Although SOD dysfunction leads to a loss of antioxidant capability, evidences have shown that the silencing of SOD1 in mice does not lead to neurodegenerative conditions [196]. In contrast, it has recently been reported that mutant SOD1 can disturb the amino acid biosynthesis of cells in a yeast model and mediate cellular destruction, accounting for the neural degeneration in ALS [197]. In addition, the reduction of the mitochondrial ROS in neurons of a double UCP-SOD1 transgenic mouse model did not recover mitochondrial function and accelerated the progression of the disease [198]. The activity of SOD was also found to reduce in red blood cells from ALS patients [199], while SOD1 activity in the CSF showed conflicting results [200, 201].

Mitochondrial oxidative damage has also been demonstrated in patients affected by sALS [202] and in a transgenic mouse model expressing a fALS-linked mutant Cu/Zn SOD1 [203]. The spinal cord and motor cortex showed increased levels of protein carbonyls, nitrosative stress, and NOS [204, 205] suggesting selectivity about protein oxidation and nitration in ALS. Mutations of other genes associated with ALS, such as TDP-43, FUS/TLS, and p62, were also found to increase mitochondrial ROS and oxidative stress [206, 207]. In addition, exogenous-added TDP-43 aggregates were found to accumulate in the cytosol of neuronal cells causing intracellular ROS production [208].

Modifications in HNE-bound proteins have been detected in ventral horn motor neurons [209], and high free HNE levels were found high in CSF and serum of ALS patients [210, 211] suggesting a diffusion of HNE from the brain to the periphery. Proteomic analysis on the spinal cord of an ALS mouse model and in CSF [212] revealed an increase in lipid peroxidation, which was also found to increase the levels of MDA-protein adducts in the lumbar spinal and cervical cord before the onset of clinical motor signs in a murine model of ALS [213]. Despite acrolein-protein adducts were not detectable in the spinal cord of ALS patients, free MDA detection has been also confirmed in ALS subjects [205].

Glycation was first detected in the spinal cord and brain samples of both sALS and fALS [214, 215] as well as in SOD1 transgenic mice. Surprisingly, the levels of soluble RAGE were found lower in serum of ALS patients with respect to control subjects [216]. From a mechanistical point of view, *in vitro* glycation affected negatively the structure and the activity of SOD1 [217]. The roles of mRNA and DNA oxidation were showed for the first time in a mouse model of ALS, suggesting the presence of an early event before the degeneration of the motor neurons and appearance of all the symptoms. ALS patients presented an increase of nuclear 8-OHdG in the motor cortex and 10-fold higher in the spinal cord tissue [205], as well as in plasma, urine, and CSF, compared to healthy people [218].

In addition to SOD1, other antioxidants showed changes in level and activity in peripheral tissues or CFS. Anyway, the modification of GSH, GPx, and GR activities appears fluctuating in the analyzed samples, suggesting a grade of variability [219], together with the variability of the pathogenic mechanisms that both lead to a fluctuation in the antioxidant profiles of ALS patients [220]. In some studies, the levels and the activities of these enzymes were found to decrease in erythrocytes [221] and in the motor cortex [222] from ALS patients. However, in another study, the GSSG/GSH ratio was found to decrease [223], and the GPx level and the GR activity were enhanced in erythrocytes, serum, and CSF [201, 224, 225]. Moreover, other studies showed that GR activity in red blood cells from ALS patients did not change, whereas CAT levels and activity were diminished [201, 224]. In addition, plasma levels of nonenzymatic antioxidants were inconsistent, with some studies showing elevated levels [226] and other with no change [227] proving again the grade of variability of the disease.

## 8. Mitochondrial Dynamics and Neurodegeneration

**Figure 3:**
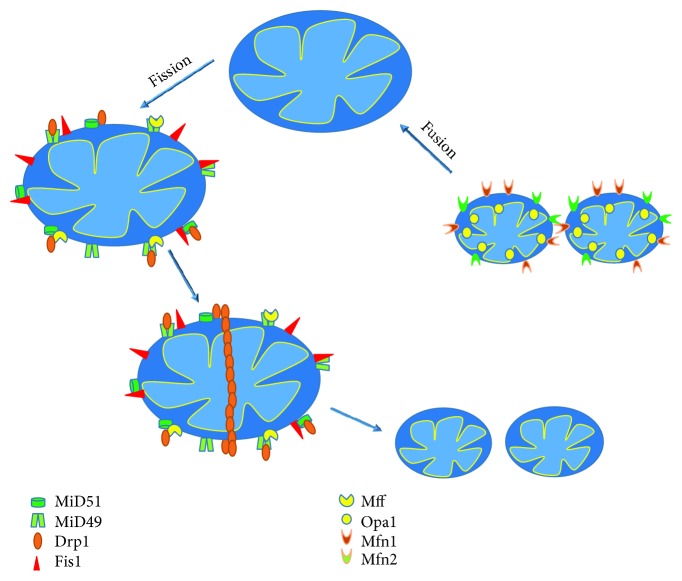
Schematic representation of mitochondrial dynamics. Drp1 in mitochondrial outer membrane forms chains promoting fission. The chain is stabilized by MiD49-Mff/Fis1 and MiD51-Mff/Fis1 complexes. Fusion is mediated by OPA-1 in the mitochondrial inner membrane and by Mfn1 and Mfn2 in the mitochondrial outer membrane. Drp1: dynamin-related protein-1; Fis1: mitochondrial fission protein 1; Mff: mitochondrial fission factor; MiD49: mitochondrial dynamics proteins of 49 kDa; MiD51: mitochondrial dynamic proteins of 51 kDa; OPA-1: optic atrophy 1; Mfn1: mitofusins 1; Mfn2: mitofusins 2.

Mitochondria are organelles with high mobility inside the cells. They can change size, morphology, and position and can also suffer fission and fusion. Fission is the process to obtain two or more daughter mitochondria from the division of a single. Fusion is the opposite: the union of two or more mitochondria to form a unique structure [228] (Figure 3). Fission and fusion are normal processes that occur continuously in many cell types. Fission is facilitated by Drp1, a protein with GTPase activity which in the mitochondrial outer membrane forms chains promoting the mitochondrial division. The chain is stabilized by MiD49 and MiD51 proteins that previously form a complex with Mff and Fis1 (Figure 3). Fusion is mediated by OPA-1 which controls the mitochondrial inner membrane fusion and by Mfn1 and Mfn2 which control the mitochondrial outer membrane fusion [229] (Figure 3).

Mitochondrial dynamics were found to be impaired in neurodegeneration. In particular, the AD brains showed abnormal expression of mitochondrial fusion and fission proteins [230], but the results are controversial. Indeed, Wang et al. reported that levels of Opa1, Drp1, Mfn1, and Mfn2 are significantly decreased, whereas the levels of Fis1 increased in the hippocampus of AD patients [231]. However, another study found increased levels of Fis1 and Drp1 and decreased levels of Mfn1, Mfn2, and Opa1 in the AD frontal cortex [232]. Moreover, the knock-in mouse Drp1+/- crossed with a mouse model of AD exhibited improved mitochondrial function [233].

Mitochondrial fission and fusion were also found to be altered in PD patients, with increased levels of p-Drp1 in white cells [234]. Moreover, *α*-synuclein induced the inhibition of mitochondrial fusion [235] by interacting with outer membrane lipids. Experiments performed in Drosophila showed that Parkin and PINK1 regulate mitochondrial dynamics in a Drp1-depending manner [236]. Both proteins are implicated in mitophagy [237] and also in mitochondrial distribution in axons [238]. DJ-1, another important PD-related protein, also regulates mitochondrial fission as consequence of a race in ROS production and its effects can be reversed by overexpression of Parkin and PINK1 [239]. Moreover, LRRK2 was also found to change Drp1 leading to mitochondrial fragmentation [240].

Lastly, mitochondrial dynamics were also altered in ALS. Indeed, an increase in mitochondrial fission is due to excessive Drp1 levels in the ALS models [241]. Moreover, changes in the levels of Fis1, Mfn1, OPA1, and Drp1 preceded motoneuron loss and symptom onset in SOD1 mutant [242]. Mitochondrial fragmentation was also found to increase both in mutant TDP-43 and FUS, and the expression of mitochondrial fission and fusion regulators appeared modified [243, 244]. Moreover, the inactivation of Drp1 or Mfn2 prevented the deficits in mitochondrial trafficking in motoneurons of mutant SOD1 or TDP-43 [243, 245].

Taking into account that mitochondrial fission and fusion proteins regulate the assembly of respiratory complexes, the direct involvement of mitochondrial fission and fusion dynamics in mitochondrial bioenergetics could be central [246]. Therefore, it is reasonable to point out that altered mitochondrial fission and fusion is probably a mechanism leading to mitochondrial dysfunction in neurodegeneration.

## 9. Conclusion

Neurodegenerative diseases are becoming increasingly prevalent in our aged populations, thus representing a primary health problem especially for this age group [4, 247]. Tremendous efforts have been already made to identify neuropathological, biochemical, and genetic biomarkers of the diseases for a diagnosis at earlier stages.

In the past thirty years, an extensive research has been performed to understand the role of mitochondria and oxidative stress not only in physiological aging, but also in neurodegenerative diseases. The whole outcome clearly affirms that both processes get impaired during aging and are established features significantly involved in the progress, if not the onset, of neurodegenerative disorders. In this moment, it is still not clear if mitochondrial dysfunction and oxidative stress could be used as markers for an early detection of aging dysfunctions or be a valid therapeutic target. However, a better knowledge of the mechanism involving mitochondria and oxidative stress in the aging process and neurodegeneration may elicit new strategies for improving the quality of life of the elderly and it would have a positive impact on the entire modern society.
